# Dipstick Spot urine pH does not accurately represent 24 hour urine PH measured by an electrode

**DOI:** 10.1590/S1677-5538.IBJU.2015.0071

**Published:** 2016

**Authors:** Mohamed Omar, Carl Sarkissian, Li Jianbo, Juan Calle, Manoj Monga

**Affiliations:** 1 Glickman Urological & Kidney Institute – Cleveland Clinic Foundation, Cleveland, OH, USA

**Keywords:** Urinary Tract, Electrodes, Urine

## Abstract

**Objectives:**

To determine whether spot urine pH measured by dipstick is an accurate representation of 24 hours urine pH measured by an electrode.

**Materials and Methods:**

We retrospectively reviewed urine pH results of patients who presented to the urology stone clinic. For each patient we recorded the most recent pH result measured by dipstick from a spot urine sample that preceded the result of a 24-hour urine pH measured by the use of a pH electrode. Patients were excluded if there was a change in medications or dietary recommendations or if the two samples were more than 4 months apart. A difference of more than 0.5 pH was considered an inaccurate result.

**Results:**

A total 600 patients were retrospectively reviewed for the pH results. The mean difference in pH between spot urine value and the 24 hours collection values was 0.52±0.45 pH. Higher pH was associated with lower accuracy (p<0.001). The accuracy of spot urine samples to predict 24-hour pH values of <5.5 was 68.9%, 68.2% for 5.5 to 6.5 and 35% for >6.5. Samples taken more than 75 days apart had only 49% the accuracy of more recent samples (p<0.002). The overall accuracy is lower than 80% (p<0.001). Influence of diurnal variation was not significant (p=0.588).

**Conclusions:**

Spot urine pH by dipstick is not an accurate method for evaluation of the patients with urolithiasis. Patients with alkaline urine are more prone to error with reliance on spot urine pH.

## INTRODUCTION

The urinary pH is an integral part of the metabolic workup of patients with nephrolithiasis, and can help direct management approaches to stone prevention.

The value of urine pH may vary according to the type of urine sample and the method of measurement. Urine pH may be measured by various ways. In the outpatient setting, two common approaches are dipstick testing and the use of a pH electrode. The pH electrode is regarded as the gold standard method of spot-urine assessment of pH (1) however, dipstick measurements offer the advantages of point-of-care assessments, home-monitoring, easy handling, and convenient cost.

To date, 24-hour urine collections are the “gold standard” for metabolic evaluation in urinary stone disease (2). However, it is time consuming and inconvenient, especially for working patients who constitute a big proportion of stone-formers. Such inconvenience may also impact patient motivation for completing repeat 24-hour collections that are believed to be necessary for accurate monitoring of response to dietary or medical interventions (3).

Our aim was to determine whether spot urine pH values measured by a dipstick are accurate to represent the 24 hours urine pH measured by an electrode, for evaluation and monitoring of patients with urolithiasis.

## MATERIALS AND METHODS

We retrospectively identified patients who presented to the urology stone clinic and had spot urine that was taken when the patient presented for a scheduled clinic visit. The 24 hour urine was collected at home by the patient, within 4 months from the spot urine date (Litholink Corp, Chicago, Il). Patients with a documented UTI at time of urine collections or those receiving urine acidifier or alkalizer medications were excluded.

To evaluate effects of spot urine results on clinical management, the accuracy of spot urine pH for predicting 24 hours pH was defined as such that a difference of more than 0.5 pH was considered a non-matching result, as dipsticks are only precise to the nearest 0.5 pH interval. For each patient, an accuracy score of a ‘yes’ or a ‘no’ was calculated by determining if the difference between the two urine samples was within 0.5 pH. To assess influence of pH values on matching accuracy, 24 hours urine pH were also grouped into clinical relevant categories, <5.5, 5.5-6.5, and >6.5 for analysis. Time interval between spot and 24 hours pH urine samples and time of day (am versus pm) the spot samples were taken were also included in the analysis.

Results were presented as means and standard deviations (SD), medians and inter quarter ranges (IQR), proportions or percentages. Group comparisons for continuous variables were done using Wilcoxon rank sum test. For categorical variables and matching rate, chi-squared test was used. Bonferroni correction was used for multiple comparisons.

In order to evaluate factors influencing rate of matching between the two pH measures, logistic regression analysis was used to model the accuracy score as defined above. Odd ratio and its 95% CI were also estimated for a variable’s influence on accuracy. All analyses and graphics were done using the statistical software package R version 3.02 (R Development Core Team, www.r-project.org). All statistics were considered significant at the level of α=0.05.

## RESULTS

A total of 600 patients were retrospectively identified who had a spot urine sample within 4 months of a 4-hour urine evaluation; the median time period between spot pH evaluation and 24-hour urine collection was 31 days (IQR 15-61). 63% (377/600) spot pH samples were taken before noon with both AM and PM samples had a mean of 6.1 pH. The average (SD) spot pH was 6.1 (0.67) compared to 6.1 (0.58) for 24 hours samples. The mean difference in pH between individual spot urine values and 24 hours collections was 0.52±0.45 pH.

**Figure 1:**
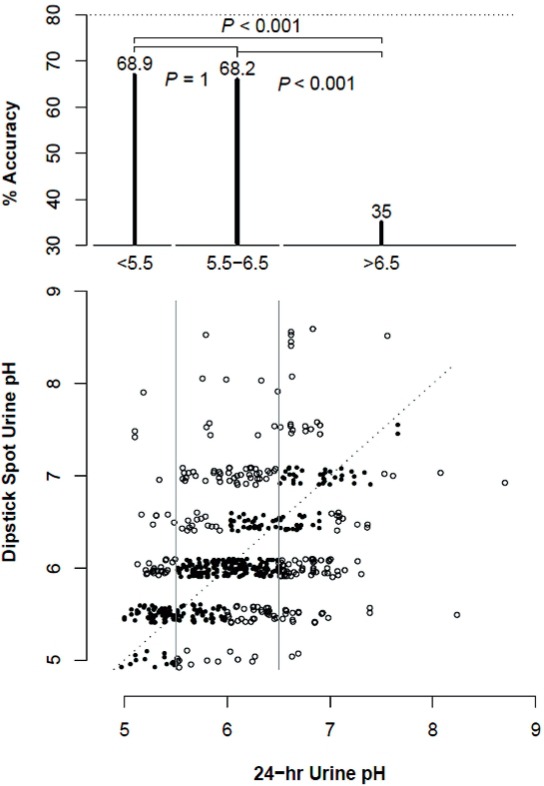
24 hours Urine pH.

The accuracy of spot urine samples to predict 24 hours pH values of <5.5 was 68.9% (71/103). The accuracy of spot urine samples to predict 24 hours pH values of 5.5-6.5 was 68.2% (232/340), while the accuracy of spot urines to detect 24 hours pH values >6.5 was only 35% (55/157). The >6.5 pH group had accuracy significantly lower than both the <5.5 pH and the 5.5-6.5 pH groups (both p’s <0.001) (Figure-1). The overall accuracy was 59.7% (358/600), which is significantly lower than an adequate accuracy of 80% (p<0.001).

Multivariable logistic regression analysis revealed that higher 24 hours urine pH was not matched by higher spot urine pH. pH >6.5 was only 19% as likely to be matched than for pH <5.5 (OR=0.19; 95% CI: 0.11-0.33; p<0.001). 24 hours urine samples and spot urine samples taken close to one day (shorter time interval) were more likely to match. Samples taken more than 75 days apart only matched 49% of the time compared to those taken in shorter intervals (OR=0.49; 95% CI: 0.32-0.77; p<0.001). Evaluating an AM or PM spot urine had no impact on the accuracy of the measurement compared to the 24-hour urine pH (OR=1.1; 95% CI: 0.78-1.57; p=0.588).

## DISCUSSION

Variations in urine pH are considered one of the well-known risk factors for urolithiasis. Monitoring urine pH has become an essential tool in the prevention and treatment protocols for stone formers. Simplifying the method of pH measurement and urine collection would make the follow-up easier and likely increase patient compliance by providing the opportunity for continuous home monitoring.

The 24 hour urine pH has been proposed to be more representative of a patient’s stone risk, avoiding the possibility of diurnal variation or circadian rhythm in urinary acidity occurring with spot urine pH (4). Urinary acid–base parameters follow diurnal patterns and it is thought these changes are due to periodic surges in gastric acid secretion.

Our goal was to evaluate the accuracy of using spot urine pH as an alternative to 24 hour urine pH, which is considered the gold standard for metabolic evaluation of stone disease.

The most clinically relevant pH values for stone formers are between 2 categories: <5.5 which is usually present with uric acid stone patients, to whom point alkalinization therapy might be initiated to decrease the risk of uric acid and calcium oxalate supersaturation, and >6.5 where either citrate supplementation may be decreased or concerns for calcium phosphate or struvite supersaturation may arise (5). We therefore focused on these cut-off points of pH to evaluate the accuracy in these ranges.

Our results suggest that the accuracy of a spot urine pH varies depending on the value of the pH a patient had at the time of measurement. Greater accuracy was noted for pH values <5.5 and 5.5-6.5 than those >6.5. One might conclude that spot urine values may be of benefit to help patients tailor their citrate intake to raise their urine pH above the 5.5 threshold, however greater reliance on 24-hour urine evaluations is warranted to avoid over-alkalinization once the pH has been increased above 6.5.

The timing of spot urine pH evaluation had no impact on the accuracy of the evaluation. Future investigation will focus on the accuracy of daily home pH monitoring with weekly averaging of the values.

The measurement tools for determining pH (dipstick versus electrode) as well as the urine collection method (one-time spot urine sample versus 24 hours collection) and the average days between spot urine samples versus 24 hours collection are likely to affect the outcome of the pH value.

A similar study by Tsong et al. (2013) reported that urine dipstick measurement had an approximately 1 in 4 (25%) risk of producing clinically significant difference (pH differences >0.5 pH unit) from meter values (6). The accuracy of pH electrode over the dipstick is well established by many researchers (7), but the difference between 24 hour and spot urine sample pH was, to our best knowledge, never been evaluated.

Though the timing of the spot pH did not coincide specifically with the date of 24-hour urine collection, we believe timing of evaluation mimics the common clinical practice of patient self-monitoring of pH levels at home in between 24-hour urine collections.

One limitation of our study is that the timing of the spot urine samples was linked to the patient’s outpatient clinic visit; as such we were unable to evaluate the utility of a first AM void or an evening sample as a screening of therapeutic pH level.

## CONCLUSIONS

We suggest that the spot urine pH by dipstick is not an accurate and dependable method for evaluation of the patients with urolithiasis. Spot pH urine evaluations are most accurate in patients with acidic urine. Its credibility should be reinforced periodically with the 24 hour electrode measured pH to avoid the high risk of errors, related to both the method of measuring and the sample used.

## References

[1] Desai RA, Assimos DG (2008). Accuracy of urinary dipstick testing for pH manipulation therapy. J Endourol.

[2] Ogawa Y, Yonou H, Hokama S, Oda M, Morozumi M, Sugaya K (2003). Urinary saturation and risk factors for calcium oxalate stone disease based on spot and 24-hour urine specimens. Front Biosci.

[3] Tiselius HG (2006). Patients’ attitudes on how to deal with the risk of future stone recurrences. Urol Res.

[4] Ayres JW, Weidler DJ, MacKichan J, Wagner JG (1977). Circadian rhythm of urinary Ph vin man with and without chronic antacid administration. Eur J Clin Pharmacol.

[5] Xu XJ, Wan MH, Ouyang JM (2009). Effect of urinary pH value on the composition of urinary nanocrystals. Guang Pu Xue Yu Guang Pu Fen Xi.

[6] Kwong T, Robinson C, Spencer D, Wiseman OJ, Karet Frankl FE (2013). Accuracy of urine pH testing in a regional metabolic renal clinic: is the dipstick accurate enough?. Urolithiasis.

[7] Khandalavala J, Van Geem TA (1999). Evaluating vaginal pH. Accuracy of two comercial pH papers in comparison to a hand-held digital pH meter. J Reprod Med.

